# Accuracy of Paris 2016 System for Non-invasive Diagnosis Bladder Malignancy

**DOI:** 10.30699/IJP.2022.548036.2833

**Published:** 2022-08-13

**Authors:** Azadeh Rakhshan, Esmat Arvin, Sam Alahyari, Behrang Kazeminegad, Tahmineh Mollasharifi, Alireza Bagheri, Fereshteh Aliakbari, Seyed Jalil Hosseini, Mohammad Soleimani, Mahsa Ahadi, Elena Jamali, Afshin Moradi, Zahra Sadeghzadeh, Saleh Ghiasi, Malihe Nasiri, Farzad Allameh

**Affiliations:** 1 *Men's Health and Reproductive Health Research Center, Shahid Beheshti University of Medical Sciences, Tehran, Iran*; 2 *Faculty of Medicine, Shahid Beheshti University of Medical Sciences, Tehran, Iran*; 3 *Department of Pathology, Faculty of Medicine, Shahid Beheshti University of Medical Sciences, Tehran, Iran*; 4 *Department of Urology, Faculty of Medicine, Shahid Beheshti University of Medical Sciences, Tehran, Iran*; 5 *Department of Biostatics, Shahid Beheshti University of Medical Sciences, Tehran, Iran*

**Keywords:** Paris system, Sensitivity, Specificity, Urine cytology

## Abstract

**Background & Objective:**

The Paris System for Reporting Urinary Cytology (TPS) is a new method for evaluating urinary cytology designed to reduce unreproducible reports. The aim of this study was to reclassify and compare urinary cytology reports with TPS criteria to determine the frequency of unreproducible reports compared to the previous system.

**Methods:**

In this study, the laboratory electronic registration system analyzed patients' urine samples taken by voided or washing and brushing methods. The cytological evaluation was performed considering the previous system and TPS by a pathologist. The results of the two systems were compared, and the sensitivity and specificity of TPS were calculated.

**Results:**

Urine samples were taken from 876 patients. The mean age of patients was 63.36 ± 12.62. Comparing the routine classification system and TPS, it was observed that the number of atypical reports in the TPS system decreased by 12%, and all of these cases were downgraded to the negative group in the new classification. The sensitivity and specificity of TPS were 29.4% and 95.1%, respectively, if suspected malignancy and positive reports for malignancy were considered. Finally, if positive reports for malignancy were selected, sensitivity and specificity changed to 11.8% and 100%, respectively.

**Conclusion:**

Although the TPS system has low sensitivity for the diagnosis of urothelial malignancies, due to its high specificity, it is possible to consider and use this classification for screening patients.

## Introduction

Bladder cancer is the second most common malignancy of the urinary tract and the second most common malignancy of middle-aged and older men. Because two-thirds of these cancers recur, careful monitoring of patients is necessary. Hematuria is the most common symptom in patients with bladder cancer. It is beneficial to distinguish the malignant causes of hematuria from its benign causes, especially through non-invasive methods (1). With various treatments including laser therapy and chemotherapy, finding a test for early detection of the disease can be lifesaving. Many laboratory tests have been utilized for diagnosis and follow-up of urological cancers like tumor markers such as PSA in prostate cancer, a biopsy of suspicious urothelial lesions, and urine cytology (2-3). Urinary cytology test is an important non-invasive method for the diagnosis and screening of new cases of urothelial carcinoma, follow-up after treatment, and recurrence Urinary cytology test is an important non-invasive method for the diagnosis and screening of new cases of urothelial carcinoma, follow-up after treatment and recurrence (4). The most important indications for urinary cytology are as follows: 1- Confirmation of the diagnosis in symptomatic patients (hematuria is the most common sign of bladder cancer). 2- Screening at-risk patients (smoking, exposure to industrial chemicals). 3- Follow-up of patients with a history of urothelial cancer (complement to cystoscopy and biopsy: discovery of small and hidden cases) (5). Urinary cytology is sensitive in diagnosing high-grade bladder tumors or carcinoma in-situ. However, even in the case of high-grade tumors, urinary cytology is a false negative in about 20% of cases. Positive cytology, even in negative cystoscopy, strongly confirms bladder cancer (6). Traditionally, a combination of cystoscopy and urinary cytology is used for follow-up every 3 months for the first 18 to 24 months after diagnosis, then every 6 months for the next two years, and then annually (7). Easy accessibility, non-invasiveness, high sensitivity and specificity for high-grade urothelial cancers and excellent impact on the evaluation of the entire urinary tract have led to the use of this method. This method can detect high-grade malignant cells in occult cancers that are not detectable by cystoscopy (8-9). Positive cytology is significantly associated with tumor recurrence and is independent of other pathoclinical variables. A positive result of this test is valuable in predicting the prognosis of primary upper urothelial cancers (10-11). Now, according to the mentioned cases, it is necessary to perform urological cytology tests concerning suspicious and susceptible patients for early detection of urothelial tumors and reduction of mortality and resulting disability. The American Society of Cytopathology, in collaboration with the International Academy of Cytology, has established a new model called The Paris System for Reporting Urinary Cytology (TPS) to standardize the interpretation of cytology specimens and prevent scattering in diagnostic criteria. In this method, a quantitative and accurate definition of the morphological criteria of cytology of malignant lesions and the criteria of urine sample adequacy is expressed for the first time (12-13). The aim of this study was to evaluate the sensitivity and specificity of the Paris 2016 method in the assessment of urine samples.

## Material and Methods

All patients who underwent urinary cytology from 2016 to 2019 were included in this multicenter study. Reasons for a urinary cytology test were as follows: 1- Symptomatic patients (hematuria is the most common symptom). 2- Patients at risk (smoking, exposure to industrial chemicals). 3- Patients with a history of urothelial cancer (complement to cystoscopy and biopsy: discovery of small and hidden cases). Exclusion criteria included insufficient sample size, excessive sediment in the urine sample, and lack of follow-up. Patients' demographic information was collected. Voided urine samples were prepared by Cytospin and stained by Papanicolaou. Urine cytology samples were analyzed using the laboratory electronic records system. Patients' urine samples were classified based on the Paris system by a team of trained pathologists for TPS. The variables evaluated concerning urine samples included the type of sampling and sample adequacy (urine volume, number of urothelial cells). The TPS cytology report was compared with the original cytological diagnoses. Patients were followed up for 4 to 30 months from the time of urinary cytology, and the cytology report was compared with the patient's pathology outcome. In this study, 2612 urine cytology samples from 876 patients were evaluated, of which 741 patients were followed up, and 135 patients were excluded from the study due to lack of follow-up.

***TPS:*** There are five categories: Negative, low-grade urothelial neoplasm (LGUN), atypical urothelial cell (AUC), suspicious high-grade urothelial carcinoma (SHGUC), and high-grade urothelial carcinoma (HGUC). The first two groups include cases with benign cytological features or mild atypical degree in a clinical context known to cause cytological changes such as poliovirus infection, kidney stones, radiation therapy, and chemotherapy. AUC was considered for non-superficial and non-degenerated urothelial cells with increased N / C ratio (> 0.5) and at least one of the following characteristics: Nuclear hyperchromasia, irregular nuclear membrane, and irregular coarse chromatin. SHGUC and HGUC cells are non-superficial, non-degenerated urothelial cells with an N / C ratio greater than 0.7, moderate to severe hyperchromasia, and at least one of the following two characteristics: Irregular mass chromatin or irregular unspecified nuclear membranes.

**Statistical Analysis**

 In this study, SPSS software version 25 (SPSS Inc., Chicago, IL., USA) was used. First, SHGUC and HGUC were considered positive reports for malignancy then susceptibility and specificity were calculated. In the next step, only HGUC was considered a positive response to malignancy, and statistical analysis was performed**.**

## Results

**Fig. 1:**
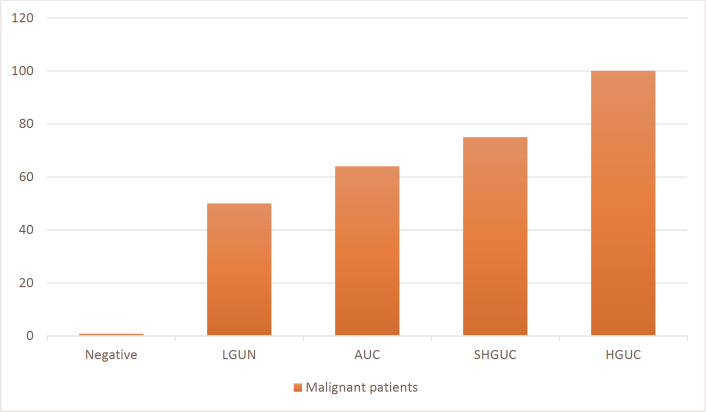
Percentage of patients who developed malignancy during follow-up in each category

The mean age of patients was 63.36±12.62 years, 559 patients were male, and 16 died. In most patients, urinary cytology indicated symptoms of suspected malignancy such as hematuria. Based on TPS, 622 (84%), 4 (0.5%), 84 (11.4%), 20 (2.8%) and 11 (1.3%) patients were classified as Negative, LGUN, AUC, SHGUC and HGUC, respectively. Table 1 shows the classification of patients' urinary cytology results in both TPS and original systems. Patients were followed up for a maximum of 3 years. All HGUC patients developed malignant lesions (bladder cancer or other malignancies such as prostate, kidney, etc.). On the other hand, among 631 patients in the negative category, only 5 patients (0.7%) experienced cancer (Figure 1).

**Table 1:** Comparison of urinary cytology results with TPS and Routine criteria

		Paris 2016 classification				
		Negative	LGUN	AUC	SHGUC	HGUC
Routine classification	Negative	612	4	0	0	**0**
	AUC	10	0	84	0	**0**
	Positive	0	0	0	20	**11**

**Comparison of Original Cytological Diagnoses with TPS**

The main difference is in the atypical category, which is 12% (10 patients) more in the original classification than in TPS.

**Follow-up**

All patients were followed up for 4 to 30 months, and urine cytology results were compared and evaluated with patients' pathology reports (Table 2). All patients in the HGUC group developed malignancy. This rate was 75% in SHGUC patients. Although the atypical category is considered a non-reproducible report, 64.3% of patients in this group developed malignancy. Also, 5 patients were diagnosed with cancer despite being in the negative group.

**Sensitivity and Specificity of TPS**

**Fig. 2:**
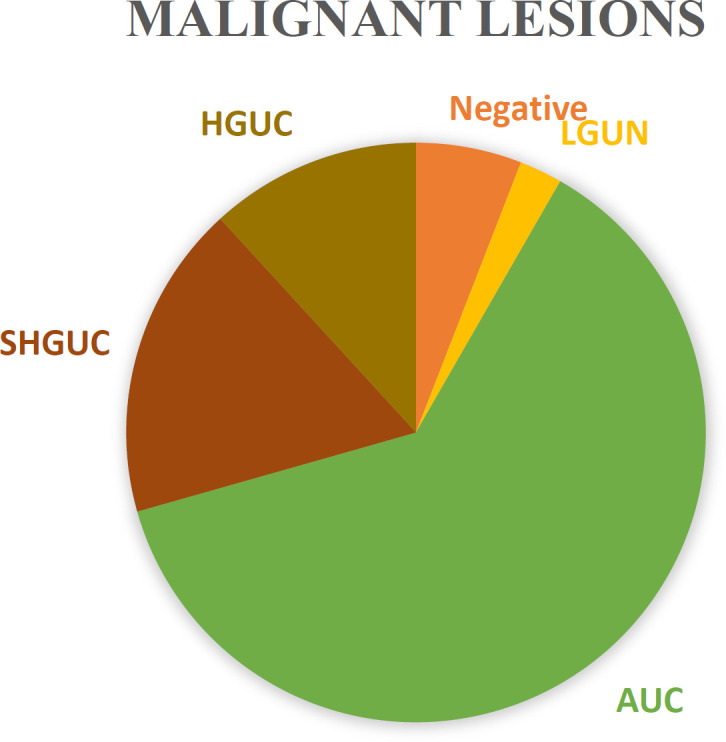
Percentage of each TPS category of total malignant patients

 First, the urinary disorders of patients were divided into two groups: malignant and non-malignant. A total of 97 patients had malignancy (bladder cancer, prostate cancer, etc.), and 644 patients had non-malignant lesions (urinary tract infection, kidney stones, etc.). Among patients with malignant lesions, 15 were reported in the SHGUC category, 10 in the HGUC category, and 54 (62.4%) were AUC (Figure 2). In this classification, the sensitivity and specificity of TPS were 29.4% and 95.1%, respectively. PPV and NPV were calculated to be 83.3% and 98.8%, respectively. In the next step, only HGUC reports were considered positive. Sensitivity and specificity were changed to 11.8% and 100%, respectively (Table 3). Finally, among 71 patients with bladder cancer, 22 were diagnosed with TPS. Therefore, sensitivity was 31%.

**Table 2:** Comparison of patients' follow-up results with TPS reports

	Follow up result	
	Cancer lesions	**Non-cancer lesions**
Negative	5	**617**
LGUN	2	**2**
AUC	54	**30**
SHGUC	15	**5**
HGUC	11	**0**

**Table 3:** Sensitivity and specificity of TPS

Results considered as positive	Sensitivity	Specificity	PPV	NPV
HGUC	11.8%	100%	100%	**89.7%**
HGUC+SHGUC	29.4%	95%	83.3%	**98.8%**

## Discussion

In this multicenter study, comparing the urinary cytology report with the Original system and TPS, it was found that the number of patients with AUC reported with TPS has decreased and reported negative. One of the most important goals of TPS was to turn atypical reports into meaningful and reproducible categories (14). Comparing the results of cytology reports of 1653 patients with the Original system and TPS, Wang* et al.* observed that the atypical report rate decreased from 18.6% to 14.4% (15). In another study of 124 patients, it was reported that by using TPS, the number of patients with atypical outcomes was reduced compared to the routine method (26% and 39%, respectively) (16). These were similar to the results of the current study. On the other hand, in a group of studies, no significant difference was observed in the atypical group in comparison TPS with the original system (17). In the study of Granados* et al.* evaluating urinary cytology of 149 patients, the number of atypical results in the Paris system was 8 times the routine classification (24.2% vs. 3%). There was also a 3-fold increase in atypical outcomes in patients with biopsy-proven non-high-grade carcinomas (18). The reason may be due to considering N / C> 0.5 as atypical in TPS, which can be found in many benign and low-grade cases. Also, after reclassification, most changes occurred in the atypical group, which became negative in the Paris system. Similar results have been reported in some other studies (19). Considering the results of various studies, it seems that the purpose of this criterion has been achieved to reduce the atypical category and turn it into more interpretable subgroups (20-21).

In this study, it was observed that most of the cytology reports of the evaluated patients were negative. In addition, 11.4%, 2.8%, and 1.3% of cytology reports were AUC, SHGUC, and HGUC, respectively. Similar results were observed in other studies (22-24). 

Following a 3-year follow-up of patients, it was observed that 100% of patients who had been classified in the HGUC category with TPS experienced urothelial cancer. This value was 75% in the SHGUC category. In a study by Ton Nu* et al.* at a 6-month follow-up of patients, out of 191 SHGUC patients, 173 had high or low-grade urothelial cancer. Also, among 256 patients with HGUC cytology reports, 238 patients developed urothelial cancer (25). At the 6-month follow-up of patients with SHGUC cytology, Piaton* et al.* found that 37.8% of them developed urothelial malignancy. However, in long-term follow-up of patients (up to 56 months), 88% of patients showed symptoms of urothelial cancer (26). Therefore, it seems that the duration of 6 months to follow patients with SHGUC cytology report is short and requires a longer time to evaluate and prevent cancer. This study's detection rate of urothelial cancer in the AUC category was 66.25%. This value has been reported in previous studies as between 8.3% -37.5% (26-29). The main reason for this heterogeneity is the difference in the follow-up time of patients for urothelial malignancies. Another reason may be the pathologist's skill in detecting atypical cells in patient specimens.

In this study, the sensitivity and specificity of TPS were calculated to be 29.4% and 95.1%, respectively. In addition, when only the HGUC result was considered positive, the sensitivity and specificity changed to 11.8% and 100%, respectively. Paula* et al.* assessed cyto-histological association in 499 patients and reported 40% and 99.3% sensitivity and specificity, respectively. In addition, if atypical were considered positive, the sensitivity and specificity would be 48.9% and 92.8%, respectively (30). Overall, the reported sensitivity of TPS ranged from 40% to 84.7%, specificity from 73% to 100%, PPV from 62.3% to 100% and NPV from 46% to 90% (31). The reason for the lower sensitivity of TPS in this study may be the low number of cancer cases compared to the total evaluated samples. In the study of Anbardar* et al.,* after considering HGUC as a positive result instead of HGUC + SHGUC, sensitivity decreased and specificity increased (32). These results are in line with the findings of the current study. The main limitation in the current study is the variability of patients' follow-up time in different categories, which occurred for various reasons, such as the patient not following the treatment, continuing treatment in another treatment center, etc. 

## Conclusion

In this study, it was observed that atypical cases in urinary cytology with TPS were reduced compared to routine criteria, and therefore, reproducibility was increased. The sensitivity and specificity of the TPS criteria were 29.4% and 95%, respectively. However, after considering HGUC as positive, these values changed to 11.8% and 100%, respectively.

## Conflict of Interest

The authors declared no conflict of interest.

## Funding

This research received no specific grant from any funding agency in the public, commercial, or not-for-profit sectors.

## References

[1] Comploj E, Trenti E, Palermo S, Pycha A, Mian C (2015). Urinary cytology in bladder cancer: why is it still relevant?. Urologia.

[2] Sirousbakht S, Rezakhaniha B (2018). Effect of colonoscopy on prostate-specific antigen; new words about an old subject. Int J Cancer Manag.

[3] Rezakhaniha B, Arian Pour N, Siroosbakhat S (2010). Effect of Cystoscopy on Prostate-Specific Antigen, New Words about Old Subject. Int J Cancer Manag.

[4] Chou R, Selph SS, Buckley DI, Gustafson KS, Griffin JC, Grusing SE, Gore JL (2016). Treatment of muscle‐invasive bladder cancer: a systematic review. Cancer.

[5] Allameh F, Sangian A, Razaghi M, Razzaghi Z, Alahyari S, Amini A Comparison of various types of lasers and transurethral resection in the treatment of bladder tumors: a systematic review and meta-analysis. Lasers Med Sci.

[6] Mitra S, Chatterjee D, Gupta K, Dass Radotra B, Dey P (2016). Urine cytology of urothelial carcinoma with villoglandular differentiation. Cytopathology.

[7] Schrag D, Hsieh LJ, Rabbani F, Bach PB, Herr H, Begg CB (2003). Adherence to surveillance among patients with superficial bladder cancer. J Natl Cancer Inst.

[8] Pan T, Lehman E, Raman JD (2021). Performance characteristics of urinary cytology in patients presenting with gross and microscopic hematuria. Am J Clin Exp Urol.

[9] Khayamzadeh M, Aliakbari F (2021). Five-year Survival Rate of Bladder Cancer in Iran during 2001-2007. Iran J Pathol.

[10] Guo A, Wang X, Gao L, Shi J, Sun C, Wan Z (2014). Bladder tumour antigen (BTA stat) test compared to the urine cytology in the diagnosis of bladder cancer: A meta-analysis. Can Urol Assoc J.

[11] Yafi FA, Brimo F, Steinberg J, Aprikian AG, Tanguay S, Kassouf W (2015). Prospective analysis of sensitivity and specificity of urinary cytology and other urinary biomarkers for bladder cancer. Urol Oncol.

[12] Barkan GA, Wojcik EM, Nayar R, Savic-Prince S, Quek ML, Kurtycz DF, Rosenthal DL (2016). The Paris System for Reporting Urinary Cytology: The Quest to Develop a Standardized Terminology. Adv Anat Pathol.

[13] Fankhauser CD, Waisbrod S, Fierz C, Becker AS, Kranzbühler B, Eberli D, Sulser T, Mostafid H, Hermanns T (2021). Diagnostic accuracy of ultrasonography, computed tomography, cystoscopy and cytology to detect urinary tract malignancies in patients with asymptomatic hematuria. World J Urol.

[14] Cowan ML, VandenBussche CJ (2018). The Paris System for Reporting Urinary Cytology: early review of the literature reveals successes and rare shortcomings. J Am Soc Cytopathol.

[15] Wang Y, Auger M, Kanber Y, Caglar D, Brimo F (2018). Implementing The Paris System for Reporting Urinary Cytology results in a decrease in the rate of the “atypical” category and an increase in its prediction of subsequent high‐grade urothelial carcinoma. Cancer cytopathology.

[16] Hassan M, Solanki S, Kassouf W, Kanber Y, Caglar D, Auger M, Brimo F (2016). Impact of implementing the Paris System for Reporting Urine Cytology in the performance of urine cytology: a correlative study of 124 cases. Am J Clin Pathol.

[17] Meilleroux J, Daniel G, Aziza J, d'Aure DM, Quintyn‐Ranty ML, Basset CM, Evrard SM, Courtade‐Saidi MM (2018). One year of experience using the Paris system for reporting urinary cytology. Cancer Cytopathology.

[18] Granados R, Duarte JA, Corrales T, Camarmo E, Bajo P (2017). Applying the Paris System for Reporting Urine Cytology increases the rate of atypical urothelial cells in benign cases: a need for patient management recommendations. Acta Cytol.

[19] Bakkar R, Mirocha J, Fan X, Frishberg DP, de Peralta-Venturina M, Zhai J, Bose S (2019). Impact of the Paris system for reporting urine cytopathology on predictive values of the equivocal diagnostic categories and interobserver agreement. Cytojournal.

[20] Brimo F, Vollmer RT, Case B, Aprikian A, Kassouf W, Auger M (2009). Accuracy of urine cytology and the significance of an atypical category. Am J Clin Pathol.

[21] Wang Y, Auger M, Kanber Y, Caglar D, Brimo F (2018). Implementing The Paris System for Reporting Urinary Cytology results in a decrease in the rate of the “atypical” category and an increase in its prediction of subsequent high‐grade urothelial carcinoma. Cancer cytopathology.

[22] Vlajnic T, Gut A, Savic S, Bubendorf L (2020). The Paris System for reporting urinary cytology in daily practice with emphasis on ancillary testing by multiprobe FISH. J Clin Pathol.

[23] Rai S, Lali BS, Venkataramana CG, Philipose CS, Rao R, Prabhu GL (2019). A quest for accuracy: evaluation of the Paris system in diagnosis of urothelial carcinomas. J Cytol.

[24] Rohilla M, Singh P, Rajwanshi A, Gupta N, Srinivasan R, Dey P, Kakkar N (2018). Cytohistological correlation of urine cytology in a tertiary centre with application of the Paris system. Cytopathology.

[25] Ton Nu TN, Kassouf W, Ahmadi‐Kaliji B, Charbonneau M, Auger M, Brimo F (2014). The value of the “suspicious for urothelial carcinoma” cytology category: a correlative study of 4 years including 337 patients. Cancer cytopathology.

[26] Piaton E, Decaussin‐Petrucci M, Mege‐Lechevallier F, Advenier AS, Devonec M, Ruffion A (2014). Diagnostic terminology for urinary cytology reports including the new subcategories ‘atypical urothelial cells of undetermined signi-ficance’(AUC‐US) and ‘cannot exclude high grade’(AUC‐H). Cytopathology.

[27] Rosenthal DL, VandenBussche CJ, Burroughs FH, Sathiyamoorthy S, Guan H, Owens C (2013). The Johns Hopkins Hospital template for urologic cytology samples: part I-creating the template. Cancer Cytopathol.

[28] Mokhtar GA, Al-Dousari M, Al-Ghamedi D (2010). Diagnostic significance of atypical category in the voided urine samples: a retrospective study in a tertiary care center. Urol Ann.

[29] Brimo F, Vollmer RT, Case B, Aprikian A, Kassouf W, Auger M (2009). Accuracy of urine cytology and the significance of an atypical category. Am J Clin Pathol.

[30] de Paula R, Oliveira A, Nunes W, Bovolim G, Domingos T, De Brot L, Bezerra S, Cunha I, Morini M, Saieg M (2020). Two‐year study on the application of the Paris system for urinary cytology in a cancer Centre. Cytopathology.

[31] Pastorello RG, Barkan GA, Saieg M (2021). Experience on the use of The Paris System for Reporting Urinary Cytopathology: review of the published literature. J Am Soc Cytopathol.

[32] Anbardar MH, Monjazeb R (2020). Reclassification of urinary cytology regarding The Paris System for Reporting Urinary Cytology with cytohistological correlation demonstrates high sensitivity for high‐grade urothelial carcinoma. Diagn Cytopathol.

